# Medical Department of Louisville University

**Published:** 1849-12

**Authors:** 


### THE WESTERN JOURNAL.

THIRD SERIES.—VOL. IV.—NO. VI.

LOUISVILLE, DECEMBER 1, 1849.

### MEDICAL DEPARTMENT OF LOUISVILLE UNIVERSITY.

The present class of medical students in this city is one of the largest that has ever assembled in the West, and the Professors are laboring in their vocations in a most acceptable manner. The Hospital facilities are far beyond any that the students have ever enjoyed in this city, and the clinical teaching fully justifies all that we predicted it would be.—Good clinical instruction must be the foundation of all useful and successful teaching, and we know of no place in the country where a real student may more fully accomplish all he purposes of medical study than in this city. We owe it to frankness to say that we have not always entertained the highest opinion of medical teaching in this city, but it gives us much more gratification to speak in terms of commendation, than it ever gave us to complain.

The great improvement of which we speak is manifested strongly in the conduct of the class of students. As a general rule, we have never known a class of young men more thoroughly awake to the teachings of the lecturers, and instead of betting upon the monotonous prescriptions of the clinical teacher, and upon the number of hours the patient would live under the treatment, they now listen attentively to a style of clinical instruction that made Dupuytren renowned throughout the world.

